# RAAS inhibitors are associated with a better chance of surviving of inpatients with Covid-19 without a diagnosis of diabetes mellitus, compared with similar patients who did not require antihypertensive therapy or were treated with other antihypertensives

**DOI:** 10.3389/fendo.2023.1077959

**Published:** 2023-01-19

**Authors:** Mykola Khalangot, Nadiia Sheichenko, Vitaly Gurianov, Tamara Zakharchenko, Victor Kravchenko, Mykola Tronko

**Affiliations:** ^1^ Endocrinology, Shupyk National Healthcare University of Ukraine, Kyiv, Ukraine; ^2^ Epidemiology of Endocrine Diseases, V. P. Komisarenko Institute of Endocrinology and Metabolism, Kyiv, Ukraine; ^3^ Infectious Diseases Hospital, Kostiantynivka, Ukraine; ^4^ Healthcare Management, Bohomolets National Medical University, Kyiv, Ukraine

**Keywords:** COVID-19, RAAS inhibitors, mortality, diabetes mellitus, angiotensin-converting-enzyme inhibitors (ACEi), angiotensin II receptor blockers (ARBs)

## Abstract

**Purpose:**

The effect of renin-angiotensin-aldosterone system (RAAS) inhibitors in combination with COVID-19 and diabetes mellitus (DM) remains unknown. We assessed the risk of death in COVID-19 inpatients based on the presence or absence of DM, arterial hypertension (AH) and the use of RAAS inhibitors or other antihypertensives.

**Methods:**

The results of treatment of all adult PCR-confirmed COVID-19 inpatients (n = 1097, women 63.9%) from 02/12/2020 to 07/01/2022 are presented. The presence of DM at the time of admission and the category of antihypertensive drugs during hospital stay were noted. Leaving the hospital due to recovery or death was considered as a treatment outcome. Multivariable logistic regression analysis was used to assess the risk of death. Patients with COVID-19 without AH were considered the reference group.

**Results:**

DM was known in 150 of 1,097 patients with COVID-19 (13.7%). Mortality among DM inpatients was higher: 20.0% *vs*. 12.4% respectively (p=0.014). Male gender, age, fasting plasma glucose (FPG) and antihypertensives were independently associated with the risk of dying in patients without DM. In DM group such independent association was confirmed for FPG and treatment of AH. We found a reduction in the risk of death for COVID-19 inpatients without DM, who received RAAS inhibitors compared with the corresponding risk of normotensive inpatients, who did not receive antihypertensives: OR 0.22 (95% CI 0.07–0.72) adjusted for age, gender and FPG.

**Conclusion:**

This result raises a question about the study of RAAS inhibitors effect in patients with Covid-19 without AH.

## Introduction

The renin-angiotensin-aldosterone system (RAAS) signaling and angiotensin-converting enzyme 2 (ACE2) have been implicated in the pathogenesis of COVID-19 because ACE2 – an enzyme that physiologically counteracts RAAS activation, is a functional receptor for SARSCoV-2 – the virus responsible for the Covid-19 pandemic (1). RAAS inhibitors may increase ACE2 expression, leading to understandable concerns about their potential danger to patients with Covid-19 (2, 3). Nevertheless, circulating levels of ACE2 in serum of type 2 diabetes mellitus (DM) patients are rather low (4), so the effect of RAAS inhibitors in combination with COVID-19 and DM remains unknown. It has also been suggested that direct and indirect loss of ACE2 through binding of SARS-CoV-2 to ACE2 partially causes the systemic manifestations of COVID-19 (5). A recent observational patient register-based study revealed that prior treatment of hypertensive patients with RAAS inhibitors, rather than increasing the risk, may actually confer some protection against in-hospital mortality (6). However, the effect of RAAS inhibitors on Covid-19 outcomes was previously evaluated in comparison with the effects of other antihypertensives but not with the absence of AH (7), it remains unknown how this effect is related to the presence of DM.

## Materials and methods

The study was conducted by analyzing the archives of one of the infectious disease hospitals of Ukraine (Kostiantynivka, Donetsk region). The archive of this hospital was recently already used to analyze mortality risk factors for Covid-19 patients, treated in 2020 (8). In 2021, patients with clinical symptoms similar to Covid-19 were hospitalized at the Infectious Diseases Hospital, which provides care to 961,000 residents. Diagnosis of Covid-19, selection of patients for hospitalization, clinical examinations and treatment were performed according to relevant national standards (9), which were updated according to WHO recommendations. Details of hospitalization criteria are shown in **Supplement Table 1**. The diagnosis of Covid-19 was confirmed by PCR. Electronic dataset was developed by Komisarenko Institute of Endocrinology and Metabolism, Kyiv, Ukraine. The results of treatment of all adult PCR - confirmed COVID-19 inpatients (n = 1,097, women 64.0%) from 02/12/2020 to 07/01/2022 are presented.

Standard clinical and anthropometric characteristics of patients (fasting plasma glucose, peripheral capillary oxygen saturation (SpO2); white blood cells (WBC); body mass index (BMI); systolic and diastolic blood pressure, were measured. Arterial hypertension (AH) category was defined as systolic/diastolic blood pressure of 140/90 mm Hg and above or hypotensive treatment. BMI was determined as the body weight (kg) divided by the height (m) squared (kg/m^2^). The presence of a documented diagnosis of diabetes mellitus (based on GP/family doctor’s references and/or use of medication) at the time of admission, myocardial infarction/revascularization history, and the category of antihypertensive drugs for the treatment of hypertension during hospital stay were considered. The antihypertensive drugs were categorized as follows: RAAS inhibitors – Angiotensin-converting-enzyme inhibitors (ACEi) or angiotensin II receptor blockers (ARBs); Calcium channel blockers; Other hypotensives. Leaving the hospital due to recovery or death was considered as a dichotomous treatment outcome.

Statistical analysis has been performed using MedCalc^®^ Statistical Software version 20.110 (MedCalc Software Ltd, Ostend, Belgium; https://www.medcalc.org; 2022). Shapiro-Wilk test was used to check the law of distribution for the quantitative data. All of the variables were not normally distributed, thus median (Me) and interquartile range (Q_I_ – Q_III_) were calculated. Pairwise comparisons were performed by the Mann–Whitney test. Frequency (%) was calculated for the qualitative data. Fisher’s exact test was used to compare frequencies. Logistic regression analysis was applied to evaluate the effects of the independent variables on the risk of death. Stepwise method was used to find the best fitting model which described the relationship between the risk of death and a set of independent variables in a multifactor logistic regression model. Odds ratios (OR) with 95% confidence interval (95% CI) were calculated to evaluate the effect of risk factors on the result of treatment. Patients with COVID-19 without AH were considered the reference group. The diagnostic performance of the logistic regression models was evaluated using Receiver Operating Characteristic (ROC) curve analysis. The area under the ROC curve (AUC) and its 95% CI were calculated. In all of the tests the p value < 0.05 was considered significant.

## Results

At the time of admission to the Infectious Diseases Hospital, diabetes was known in 150 of 1,097 patients with COVID-19 (13.7%). The share of women in the group of individuals with diabetes was 68.0%, while among individuals without diabetes it was 63.4%, i.e., there is no statistically significant difference in gender distribution in these groups (p = 0.314). Glycemia and phenotypic characteristics in the diabetes group differed from those without such a diagnosis at the time of admission: moderate hyperglycemia and obesity were present in most people with diabetes. Age, FPG, BMI and blood pressure were higher in the diabetes group. The history of myocardial infarction, coronary revascularization, and stroke was also more common in the diabetes group. However, body temperature and oxygen saturation, i.e., characteristics that reflect the course of COVID-19, did not differ in these groups (**Table 1**).

**Table 1:** Some characteristics of patients with COVID-19 depending on their “diabetes mellitus” diagnosis at the time of admission to the infectious disease hospital.

Characteristics	No Diabetes n=947 (600 women)	Diabetes n=150 (102 women)	p
Age, all (yrs)	61.7 (49.5–69.7)	67.1 (60.6–71.9)	**<0.001**
Age, women (yrs)	63.0 (51.1–70.1)	67.9 (61.6–72.2)	**<0.001**
Age, men (yrs)	60.3 (44.9–69.3)	66.0 (52.2–70.7)	**0.022**
FPG (mmol/L)	5.6 (4.8–6.6)	9 (6.9–12.4)	**<0.001**
Saturation O2 (%)	89 (86–95)	92 (86–95)	0.174
Systolic BP (mmHg)	130 (120–140)	135 (130–148)	**<0.001**
Diastolic BP (mmHg)	80 (75–85)	80 (78–90)	**<0.001**
Body temperature (°C)	37.9 (37.4–38.5)	37.8 (37.2–38.5)	0.101
BMI, (kg/m^2^)^1^	29.4 (25.9–32.7)	32.7 (28.7–36.7)	**<0.001**
BMI, women (kg/m^2^)	29.2 (25.7–32.6)	33.2 (27.9–36.9)	**<0.001**
BMI, men (kg/m^2^)	29.8 (26.2–32.7)	31.8 (29.9–33.7)	**<0.001**
Stroke history	8 (0.8)	6 (4.0)	**0.007**
MI/revascularization history	6 (0.6)	6 (4.0)	**0.003**
AH	354 (37.4)	97 (64.7)	**< 0.001**

FPG, fasting plasma glucose; BMI, body mass index; BP, blood pressure; MI, myocardial infarction; AH, arterial hypertension.

Data are medians and inter quartile ranges (Q_I_-Q_III_) or n and %. The comparison was based on the Mann-Whitney test.

^1^BMI data were only for 852 “No Diabetes” patients and 130 “Diabetes” patients.

Bold values denote statistical significance at the P < .05 level.

People with diabetes were almost twice as likely to be treated with positive pressure ventilation (PPV) and mortality among them was also higher than in the group without diabetes: 20.0% *vs* 12.4% respectively. There were twice as less people without hypertension in the group with diabetes as in the group without diabetes. The use of antihypertensives also differed: ACEIs/ARBs was used by 25.3% of persons in the diabetes group and only 9.4% in the non-diabetes group. Steroid hormones for the treatment of COVID-19 were used in most patients of both groups, the structure of their use differed, but not significantly. Insulin was used to treat 37.3% of patients diagnosed with diabetes at the time of admission (**Table 2**).

**Table 2:** Distribution of some treatments and mortality of patients with COVID-19 depending on their “diabetes mellitus” diagnosis at the time of admission to the infectious disease hospital.

		No Diabetes n=947	Diabetes n=150	p
Treatment of arterial hypertension (AH)	no AH and no AH treatment	593 (62.6)	53 (35.3)	**<0.001**
	AH, no AH treatment	140 (14.8)	22 (14.7)	
	ACEIs/ARBs	89 (9.4)	38 (25.3)	
	Calcium channel blockers	38 (4.0)	20 (13.4)	
	Other hypotensives	87 (9.2)	17 (11.3)	
Insulin		–	56 (37.3)	–
Steroids	no steroids	407 (43.0)	62 (41.3)	**0.048**
	Dexamethasone	488 (51.6)	80 (53.3)	
	dexamethasone + methylprednisolone	27 (2.9)	0 (0.0)	
	methylprednisolone	24 (2.5)	8 (5.3)	
PPV		62 (6.5)	18 (12.0)	**0.026**
Lethal outcomes		117 (12.4)	30 (20.0)	**0.014**

ACEIs, Angiotensin-converting-enzyme inhibitors; ARBs, angiotensin II receptor blockers; PPV, positive pressure ventilation. Data are n and %. The comparison of the two groups was performed according to Fisher’s exact test or the chi-square test.

Bold values denote statistical significance at the P < .05 level.

Male gender, age, blood glucose, systolic blood pressure, body mass index (continued variables) were positively associated with the risk of dying in hospitalized patients with COVID-19 who were not diagnosed with diabetes mellitus. The presence of hypertension in the absence of any treatment or antihypertensive treatment with drugs that did not belong to the categories of ACEIs/ARBs or Calcium channel blockers was also associated with a risk of death. There was a statistical tendency (p = 0.060) mortality risk reduction in patients with COVID-19 taking ACEIs/ARBs. Dexamethasone treatment increased the chances of death (**Table 3**).

**Table 3:** Risks of death of COVID-19 inpatients without diabetes mellitus (n=947) estimated using one-way logistic regression models.

Factors		Model coefficient, b ± m	p	OR (95% CI)
Gender	women	Reference		
	men	0.41 ± 0.20	**0.039**	1.51 (1.02–2.23)
Age, yrs		0.060 ± 0.009	**<0.001**	1.06 (1.04–1.08)
FPG, mmol/L		0.18 ± 0.05	**<0.001**	1.20 (1.09–1.32)
Systolic BP, mm Hg		0.017 ± 0.006	**0.002**	1.02 (1.01–1.03)
Diastolic BP, mm Hg_		0.006 ± 0.010	0.522	–
BMI, kg/m^2^		0.046 ± 0.021	**0.032**	1.05 (1.00–1.09)
Treatment of arterial hypertension (AH)	no AH and no AH treatment	Reference		
	AH, no AH treatment	0.54 ± 0.27	**0.045**	1.72 (1.01–2.92)
	ACEIs/ARBs	-1.13 ± 0.60	0.060	–
	Calcium channel blockers	-0.23 ± 0.62	0.704	–
	Other hypotensives	1.63 ± 0.26	**<0.001**	5.11 (3.05–8.55)
Steroids	no steroids	Reference		
	Dexamethasone	0.74 ± 0.22	**0.001**	2.10 (1.36–3.23)
	Dexamethasone + methylprednisolone	0.95 ± 0.53	0.072	–
	Methylprednisolone	0.48 ± 0.64	0.451	–

FPG, fasting plasma glucose; BMI, body mass index; AH, arterial hypertension; ACEi, Angiotensin-converting-enzyme inhibitors; ARB’s, angiotensin II receptor blockers.

Bold values denote statistical significance at the P < .05 level.

Stepwise method (entering variable if p < 0.1, removing variable if p > 0.2) was used to find the best fitting model that described the relationship between the risk of death and a set of independent variables in a multifactor logistic regression model. Four variables were selected: gender, age, FPG and treatment of arterial hypertension (AH). The area under the curve of this model (**Supplementary Figure 1**) AUC = 0.78 (95% CI 0.75 - 0.81), indicating an average-strength association between the risk of death and four selected variables.

If gender, age, FPG, and antihypertensive categories were considered within the same model (**Table 4**), the trend became statistically significant (p = 0.013) and indicated a multiple reduction in the chances of death for patients treated with ACEIs/ARBs: OR = 0.22 (0.07–0.72). The risks associated with male gender, age, FPG, and other AH treatments did not change significantly from one-factor estimates.

**Table 4:** Analysis of a multifactor logistic regression model for predicting the risk of death for patients without diabetes.

Factors		Model coefficient, b ± m	p	OR (95% CI)
Gender	women	Reference		
	men	0.52 ± 0.23	**0.022**	1.67 (1.08–2.61)
Age, yrs		0.063 ± 0.010	**<0.001**	1.07 (1.05–2.61)
FPG, mmol/L		0.12 ± 0.05	**0.026**	1.12 (1.01–1.24)
Treatment of arterial hypertension (AH)	no AH and no AH treatment	Reference		
	AH, no AH treatment	0.23 ± 0.29	0.435	_
	ACEIs/ARBs	-1.53 ± 0.61	**0.013**	0.22 (0.07–0.72)
	Calcium channel blockers	-0.70 ± 0.64	0.274	–
	Other hypotensives	1.23 ± 0.29	**<0.001**	3.41 (1.94–5.98)

FPG, fasting plasma glucose; AH, arterial hypertension; ACEi, Angiotensin-converting-enzyme inhibitors; ARB’s, angiotensin II receptor blockers.

Bold values denote statistical significance at the P < .05 level.

In 150 patients with diabetes and COVID-19, evaluation using one-way logistic regression models confirmed a positive association between age, FPG, BMI, and antihypertensive treatment with drugs that did not belong to the ACEIs/ARBs or calcium channel blockers and the likelihood of death (**Table 5**). Within the joint model (age, FPG, AH treatment), there was no association between ACEIs/ARBs and the chances of death (**Table 6**).

**Table 5:** Risks of death of COVID-19 inpatients with diabetes mellitus (n=150) estimated using one-way logistic regression models.

Factors		Model coefficient, b ± m	p	OR (95% CI)
Gender	women	Reference		
	men	0.26 ± 0.43	0.541	–
Age, yrs		0.047 ± 0.021	**0.023**	1.05 (1.01–1.09)
FPG, mmol/L		0.11 ± 0.05	**0.018**	1.12 (1.02–1.22)
Systolic BP, mm Hg		0.017 ± 0.009	0.060	–
Diastolic BP, mm Hg		0.021 ± 0.020	0.300	–
BMI, kg/m^2^		0.094 ± 0.040	**0.020**	1.10 (1.02–1.19)
Treatment of arterial hypertension (AH)	no AH and no AH treatment	Reference		
	AH, no AH treatment	-1.32 ± 1.09	0.223	–
	ACEIs/ARBs	-1.13 ± 0.60	0.060	–
	Calcium channel blockers	0.88 ± 0.62	0.156	–
	Other hypotensives	2.08 ± 0.62	**0.001**	8.03 (2.36–27.3)
Steroids	no steroids	Reference		
	Dexamethasone	0.30 ± 0.53	0.486	–
	Methylprednisolone	-0.41 ± 1.12	0.713	–

FPG, fasting plasma glucose; BMI, body mass index; AH, arterial hypertension; ACEIs, Angiotensin-converting-enzyme inhibitors; ARBs, angiotensin II receptor blockers.

**Table 6:** Analysis of a multifactor logistic regression model for predicting the risk of death for patients with diabetes.

Factors		Model coefficient, b ± m	p	OR (95% CI)
Age, yrs		0.043 ± 0.022	0.058	1.04 (1.00–1.09)
FPG, mmol/L		0.13 ± 0.05	**0.016**	1.14 (1.02–1.26)
Treatment of arterial hypertension (AH)	no AH and no AH treatment	Reference		
	AH, no AH treatment	-1.26 ± 1.11	0.255	–
	ACEIs/ARBs	-0.26 ± 0.64	0.688	–
	Calcium channel blockers	0.71 ± 0.65	0.277	–
	Other hypotensives	1.98 ± 0.682	**0.001**	7.24 (1.93–27.2)

FPG, fasting plasma glucose; AH, arterial hypertension; ACEIs, Angiotensin-converting-enzyme inhibitors; ARBs, angiotensin II receptor blockers.

The category of “participants that were apparently hypertensive but did not get antihypertensive treatment” (AH, no AH treatment) included COVID-19 inpatients, who have not previously received antihypertensive treatment and their maximum blood pressure was often borderline for the presence of arterial hypertension: the median was equal to 140/90 mmHg and did not exceed the corresponding indicator in groups of hypotensive treatment (**Supplement Table 3**).

## Discussion

We are presenting the results of an observational study on the outcomes of all COVID-19 inpatients (n = 1097) treated in one of the infectious diseases hospitals of Ukraine in 2021.

The group of COVID-19 inpatients (n = 150) with known diabetes mellitus at the time of admission, represented within this cohort, was expected to show not only higher mortality and PPV demand, but also more frequent use of ACEIs/ARBs *vs*. inpatients without such a diagnosis. However, concerns about the detrimental effects of ACEIs/ARBs on patients with COVID-19, which were declared at the beginning of the pandemic (2), were not justified, i.e. we did not find the expected increase in mortality associated with these drugs. Moreover, we found that the use of RAAS inhibitors for the treatment of hypertension in patients with COVID-19 who did not have DM was associated with a reduced risk of death. Within the logistic regression model, which also takes into account age, sex and plasma glucose levels, the use of ACEIs/ARBs (but not other antihypertensive drugs) is associated with a significant decrease in the chances of dying: OR 0.22 (0.07–0.72), patients without AH were considered as the reference group. RAAS inhibitors did not affect the mortality of COVID-19 inpatients with DM.

In 2020, Italian researchers who observed COVID-19 outcomes in a small cohort of hypertensives (n = 133) concluded that chronic use of RAAS inhibitors does not negatively affect the clinical course of COVID-19 in hypertensive patients. A significantly lower risk of admission to intensive care was observed in COVID-19 positive subjects chronically treated with ACEIs/ARBs as compared with other hypertensive patients, whereas the rates of hospitalization, oxygen therapy, noninvasive ventilation, and death did not differ between the 2 groups (10). Recently, British researchers who analyzed outcomes of 9,197 hospitalized patients with Covid-19 informed that the use of RAAS inhibitors tended to have a protective effect for in-hospital mortality in fully adjusted models (OR 0.88, 95% CI 0.78,0.99). The variables used in these fully adjusted models included age, sex, diabetes, chronic kidney disease, chronic obstructive pulmonary disease, obesity and known heart failure (6). Chinese researchers using the propensity score-matched analysis of 1128 hospitalized COVID-19 patients with hypertension, demonstrated a lower risk of COVID-19 mortality in patients who received ACEI/ARB versus those who did not (adjusted hazard ratio, 0.37 [95% CI, 0.15–0.89] (11).

Our risk assessment model was less complex because it did not include the presence of chronic diseases other than hypertension and was implemented separately for patients without DM and with known DM. In addition, we assessed the risk of death for RAAS inhibitors not comparing with any other antihypertensive drugs as in the mentioned studies (10, 11), but in comparison with the corresponding risk for normotensive patients.

The hypothesis that RAAS inhibitors may prevent COVID-19 deaths in hospitalized, hypertensive patients has been previously published by Spanish researchers based on the SEMI-COVID-19 cohort registry data (12). This benefit seems to equalize the risk of treated patients to the risk of non-hypertensive patients. But in our study, RAAS treatment reduces the risk of death beyond the risk of normotensives as the reference group. Rodilla et al, 2020 (12) were probably the first to describe a reduction in the risk of death in hospitalized patients with COVID-19 treated with RAAS inhibitors, which does not contradict our data on the probable protective role of RAAS inhibitors. But we failed to prove the independent nature of the effect of arterial hypertension on mortality in COVID-19 inpatients. The National Cohort Study in England investigated 19,256 COVID-19–related intensive care unit admissions and revealed that patients with type 2 diabetes were at increased risk of mortality independently of hypertension (13).

Today there are still many controversies in the relationship between hypertension and COVID-19. This concerns the predictive value of hypertension, the effect of blood pressure levels, the impact of previously known and newly diagnosed hypertension, and the effect of antihypertensive therapy on the severity and outcomes in COVID-19 patients (14).

The lack of consideration of the degree of frailty/comorbidity of the participants is an important limitation of our study. The authors will try to overcome this limitation as soon as the humanitarian situation in the country normalizes. Another limitation of our study is the fact that we compared outcomes of patients, hospitalized with COVID-19 depending on whether the DM diagnosis was known prior to admission. This means that the group of COVID-19 inpatients without DM could contain persons with unknown diabetes. Stress-induced acute hyperglycemia is commonly observed in critical illness (15), therefore diagnosing DM during acute illnesses and infections is considered undesirable. Besides, the compared groups have undeniable anthropometric, biochemical, and other differences (**Table 1**) which confirms the validity of categorization we have applied. The question of qualitative sufficiency of Covid-19 inpatients with known DM group (n=150) to confirm or refute the null hypothesis (type II errors) was resolved positively (see **Supplementary Table 2** and **Supplementary Figure 3**).

Thus, we found a very significant reduction in the risk of death for hospitalized patients with COVID-19 without DM, who received RAAS inhibitors compared with the corresponding risk of normotensive COVID-19 inpatients who did not receive antihypertensive treatment. Attention should be paid to the lack of influence of RAAS inhibitors on the risk of mortality of COVID-19 inpatients with DM. A recent retrospective multicentre European study (16) revealed no association between mortality and renin-angiotensin-aldosterone system inhibitor therapy in adults with diabetes admitted to hospital with COVID-19.

We hypothesize that the decrease in circulating levels of ACE2 resulting from both DM and COVID-19 is too great for RAAS inhibitors to fully overcome, that is why the positive effect of RAAS inhibitors may be absent in patients with COVID-19. Despite the opinion that effects of RAAS inhibitors on ACE2 in humans are still uncertain (1), their treatment potential for Covid-19 is rather positive (17, 18). We believe that our results not only confirm the safety of RAAS inhibitors in hypertensive patients with Covid-19, but also raise a question about the study of their therapeutic effect in patients with Covid-19 without arterial hypertension. The long-term positive experience of using RAAS inhibitors in normotensive patients with diabetic kidney disease (19) is well known. Thus, we speculate that ACE2-based regulation strategies may become one of the most promising approaches for future therapies and improve disease prognosis in COVID-19. Thus, medications that were thought to spread the pandemic and increase mortality may have therapeutic potential not only for individuals with AH, but also normotensive patients with COVID-19. Interestingly, an almost similar situation arose with Sodium-glucose co-transporter 2 inhibitors (SGLT2i) - a new class of oral, glucose-lowering agents used for the management of type 2 diabetes (T2D). At the beginning of the COVID-19 pandemic, SGLT2i was not recommended to be included for the treatment of T2D COVID-19 patients due to fears of the development of euglycemic diabetic ketoacidosis (DKA), a potentially fatal clinical entity (20). Recently, Greek researchers (21) hypothesized that SGLT2i might have a role in the future management of COVID-19 and raised a question: do SGLT2i have the potential to improve COVID-19-related outcomes in people with or even without diabetes? To answer this question they are counting on results of two ongoing, randomized-controlled trials (RCTs) (21). It is reasonable to introduce RCTs for studying COVID-19-related effects of RAAS inhibitors as well.

## Data availability statement

The original contributions presented in the study are included in the article/**Supplementary Material**. Further inquiries can be directed to the corresponding author.

## Ethics statement

The study was approved by the ethics committee of the Institute of Endocrinology and Metabolism (National Academy of Medical Sciences of Ukraine) protocol # 35/4-KE from 01/04/2021. The patients/participants provided their written informed consent to participate in this study.

## Author contributions

MK and MT conceived the study design. NS participated in data collection. MK, NS, TZ and VK participated in data analysis and interpretation. VG carried out the final statistical analysis. All authors contributed to the article and approved the submitted version.

## Supplementary material

Supplementary Figure 1 ROC-curve of the four-factor (gender, age, FPG and treatment of AH) model of the risk of death for patients without diabetes (95% CI is presented). The criterion value is corresponding with the Youden index.

Supplementary Figure 2 ROC-curve of the three-factor model (age, FPG and treatment of AH) of the risk of death for patients with diabetes (95% CI is presented). The criterion value is corresponding with the Youden index.

Supplementary Figure 3 Central (solid line) and non-central (dotted line) distribution to protocol of power analyses.
