# Supplementary figures and images for: RAAS inhibitors are associated with a better chance of surviving of inpatients with Covid-19 without a diagnosis of diabetes mellitus, compared with similar patients who did not require antihypertensive therapy or were treated with other antihypertensives
Source: Front Endocrinol (Lausanne). 2023 Jan 19;14:1077959. doi: 10.3389/fendo.2023.1077959 (PMC9900734; doi:10.3389/fendo.2023.1077959)

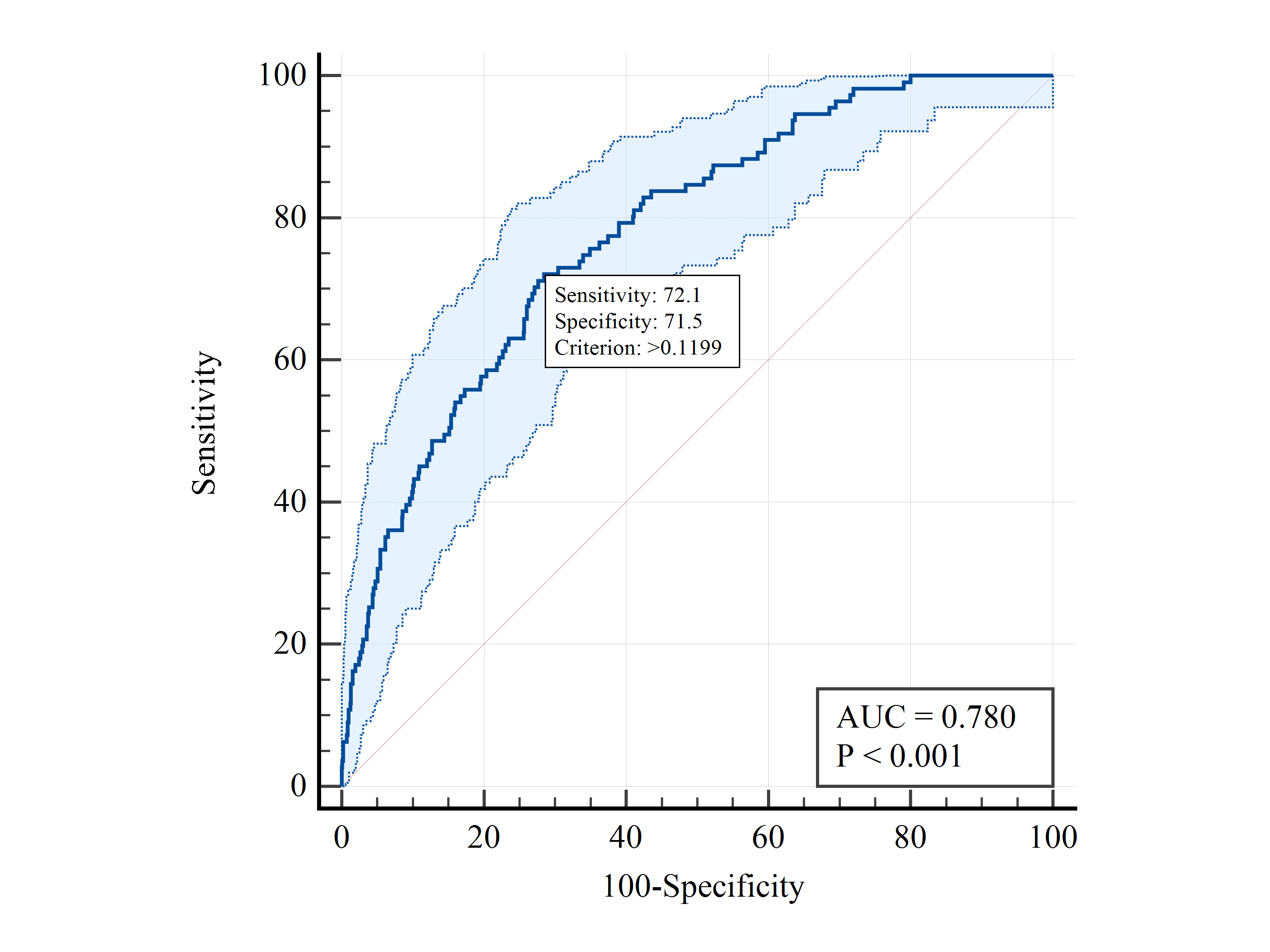

Supplement: Supplementary Figure 1 — ROC-curve of the four-factor (gender, age, FPG and treatment of AH) model of the risk of death for patients without diabetes (95% CI is presented). The criterion value is corresponding with the Youden index. [file Image_1.tif]

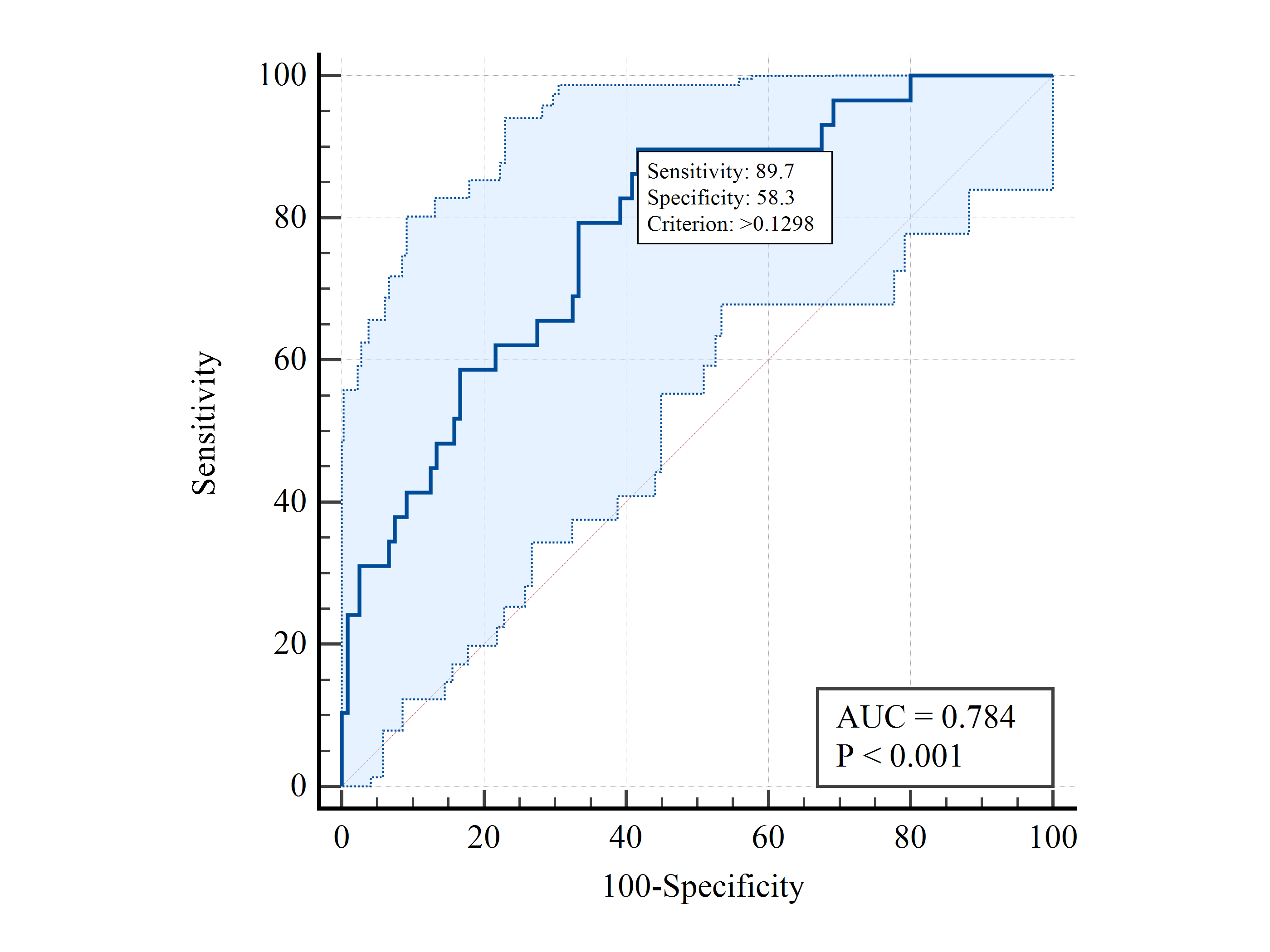

Supplement: Supplementary Figure 2 — ROC-curve of the three-factor model (age, FPG and treatment of AH) of the risk of death for patients with diabetes (95% CI is presented). The criterion value is corresponding with the Youden index. [file Image_2.tif]

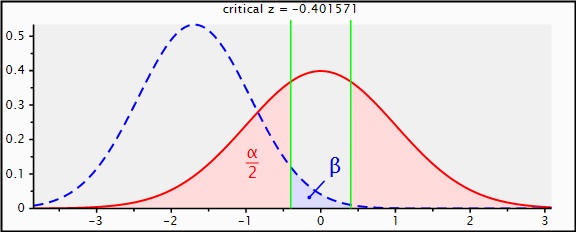

Supplement: Supplementary Figure 3 — Central (solid line) and non-central (dotted line) distribution to protocol of power analyses. [file Image_3.jpeg]
