# Supplementary material for: RAAS inhibitors are associated with a better chance of surviving of inpatients with Covid-19 without a diagnosis of diabetes mellitus, compared with similar patients who did not require antihypertensive therapy or were treated with other antihypertensives
Source: Front Endocrinol (Lausanne). 2023 Jan 19;14:1077959. doi: 10.3389/fendo.2023.1077959 (PMC9900734; doi:10.3389/fendo.2023.1077959)
Supplement: Supplementary file 4 [file Table_1.docx]

Supplement table 1

COVID-19 hospitalization criteria (extracted from 10)

Hospitalization according to clinical criteria is indicated for patients with suspected / confirmed COVID-19 in:

- moderate and severe signs of pneumonia and / or respiratory failure (increased respiratory rate above physiological norm, hemoptysis, SpO2 when measured with a pulse oximeter ≤ 93%) in the presence of radiologically confirmed pneumonia;
- availability of clinical and instrumental data of acute respiratory distress syndrome (ARDS);
- availability of clinical and laboratory data of sepsis and / or septic shock (systemic inflammatory response syndrome);
- availability of clinical and laboratory data of organ / system insufficiency other than respiratory failure;
- patients, regardless of the severity of the condition, who belong to the risk group of complications: severe hypertension, decompensated diabetes mellitus, immunosuppressive conditions, severe chronic pathology of the respiratory and cardiovascular systems, renal failure, autoimmune diseases,
- severe allergic diseases, cerebrovascular diseases in the stage of decompensation), cancer;
- patients, regardless of the severity of the condition, in which there is an increase in temperature above 38 ° C, which is difficult to correct (temporary, no more than 1-1.5 hours of reduction on the background of antipyretic drugs, followed by its increase).

10. STANDARDS OF MEDICAL CARE "CORONAVIRUS DISEASE (COVID-19)" Ministry of Health of Ukraine (Document in Ukrainian) https://moz.gov.ua/uploads/3/19713-standarti_med_dopomogi_covid_19.pdf
