# Supplementary material for: RAAS inhibitors are associated with a better chance of surviving of inpatients with Covid-19 without a diagnosis of diabetes mellitus, compared with similar patients who did not require antihypertensive therapy or were treated with other antihypertensives
Source: Front Endocrinol (Lausanne). 2023 Jan 19;14:1077959. doi: 10.3389/fendo.2023.1077959 (PMC9900734; doi:10.3389/fendo.2023.1077959)
Supplement: Supplementary file 5 [file Table_2.docx]

Suppl. table 2. Protocol of power analyses (G*Power 3.1.9.7, Faul, F., Erdfelder, E., Buchner, A., & Lang, A.-G.)

| z tests - Logistic regression | | | |
| --- | --- | --- | --- |
| Options: | Large sample z-Test, Demidenko (2007) with var corr | | |
| Analysis: | Post hoc: Compute achieved power | | |
| Input: | Tail(s) | = | Two |
|  | Odds ratio | = | 0.25 |
|  | Pr(Y=1\|X=1) H0 | = | 0.2 |
|  | α err prob | = | 0.688 |
|  | Total sample size | = | 150 |
|  | R² other X | = | 0.2 |
|  | X distribution | = | Binomial |
|  | X parm π | = | 0.25 |
| Output: | Critical z | = | -0.4015707 |
|  | Power (1-β err prob) | = | 0.960354 |
